# Retention on antiretroviral therapy and drivers of lost‐to‐follow up in the Central African Republic: a longitudinal analysis

**DOI:** 10.1002/jia2.26387

**Published:** 2024-12-05

**Authors:** Gaspard Tekpa, Jules Inikoutiyo, Christian Yonli, Celia Noguera, Pierre Prince Lujwiro, Laure Gigout, Aboubacar Hachimou, Sydney Romaric, Raphaël Mabaïlao, Marie Charlotte Banthas, Larissa Bertille Mbia, Paulette Rose Mbay, Kevin Romuald, Alain Sana, Florida Roberte, Laura Moretó‐Planas, Eric Goemaere, Calorine Mekiedje, Stella Ouanekpone, Maria Amparo Núñez‐Andrés, Sarah Hoibak, Xavier Vallès

**Affiliations:** ^1^ Service des maladies infectieuses Hôpital de l'Amitié Bangui Central African Republic; ^2^ Croix Rouge Française Bangui Central African Republic; ^3^ Ministère de la Santé et la Population de la Republique Centrafricaine Bangui Central African Republic; ^4^ Médecins Sans Frontières Operational Cell Barcelona‐Athens Barcelona Spain; ^5^ Centre for Infectious Disease Epidemiology and Research, School of Public Health and Family Medicine, Faculty of Health Sciences University of Cape Town Cape Town South Africa; ^6^ Médecins Sans Frontières Southern Africa Medical Unit Cape Town South Africa; ^7^ Médecins Sans Frontières Operational Cell Barcelona‐Athens Bangui Central African Republic; ^8^ Department of Civil and Environmental Engineering Universitat Politècnica de Catalunya‐BarcelonaTech Barcelona Spain; ^9^ The Global Fund to Fight AIDS Tuberculosis and Malaria Geneva Switzerland; ^10^ International Health Program (PROSICS), Direcció Territorial de Malalties Infeccioses Metropolitana Nord Institut Català de la Salut Badalona Spain; ^11^ Fundació Lluita contra les Infeccions Badalona Spain; ^12^ Germans Trias i Pujol Research Institute Badalona Spain

**Keywords:** Africa, ARV, health systems, HIV epidemiology, retention to care, structural drivers

## Abstract

**Introduction:**

The retention in care of patients undergoing antiretroviral therapy (ART) is a cornerstone for preventing AIDS‐associated morbidity and mortality, as well as further transmission of HIV. Adherence to ART poses particular challenges in conflict‐affected settings like the Central African Republic (CAR). The study objective was to estimate the rate of lost‐to‐follow‐up (LTFU) and determine factors associated with LTFU among patients living with HIV under ART in CAR.

**Methods:**

A retrospective cohort analysis was conducted using data from patients being managed at 42 representative ART dispensing sites (i.e. management of ≥200 patients) in the seven health regions of CAR which started ART between January 2019 to September 2021 and followed up to December 2021. The outcome of LTFU was defined as a failure of a patient to attend a scheduled ART refill appointment for at least 90 days from the last appointment. Patients were censored at the first LTFU event.

**Results:**

A total of 6844 patients enrolled in ART care were included in the analysis, of whom 67.5% were females. The mean age (standard deviation) was 35.3 years (10.5). Forty‐two per cent (*n* = 2874/6844) had an LTFU event during the follow‐up period. However, 23.2% (*n* = 666/2874) returned to care after LTFU. Overall retention in antiretroviral care at 12 months was 64.2% (CI 63.0−65.5), which ranged from 76.1% in the capital to 48.2% in the inner country region. Risk factors related to LTFU were being male (adjusted hazard ratio [aHR] 1.33; CI 1.1−1.5), age < 25 (aHR 1.46; CI 1.1−1.9), living in regions outside the capital (aHR 1.83; CI 1.6−2.3) and undernutrition (aHR 1.13; CI 1.0−1.3).

**Conclusions:**

Retention to care in CAR is suboptimal, especially in the inner country. Our results underline the difficulties involved in retaining patients in ART in complex settings, the interplay between poor retention, social unrest, stigma, food insecurity and HIV epidemic control, and the need for tailored programming and interventions like differentiated treatment strategies and complementary food provision.

## 1 INTRODUCTION

AIDS‐related mortality has dramatically declined since 2010 in middle‐ and low‐income countries, especially in sub‐Saharan Africa, where most HIV cases still occur [1, 2]. This decline in mortality has been attributed to the scale‐up of antiretroviral therapy (ART) coverage [1, 2], fostered by several and more recent strategies which have contributed to improved HIV care. These include the introduction of new testing and access‐to‐care strategies, ART delivery through differentiated service delivery and new antiretroviral (ARV) formulations, like the recent inclusion and scale‐up of dolutegravir as first‐line ART. However, in recent years, there has been an observed slowdown in key indicators of progress [1, 2]. This is due to remaining structural barriers that may not be addressed by these interventions [3]. These barriers are characteristic of populations that are harder to reach (e.g. youth, mobile populations, etc.) and/or may respond to contextual factors (geographic, social, cultural or political) that are specific in different countries and may also vary by region. Furthermore, in fragile contexts, progress can be easily set back by political or social instability, or natural disasters. Difficulty to deliver ARV may be further exacerbated by individual‐level factors. These factors include stigma, cultural beliefs or the impact to mental health of structural factors like violence and poverty (i.e. post‐traumatic stress, anxiety and depression) [4]. This scenario accurately reflects the situation in the Central African Republic (CAR), a country characterized by ongoing political and social instability with historical roots in the colonial period [5, 6]. Access to health services may be severely limited depending on the region in CAR [7]. As a result, the CAR ranks 188 among 191 countries in the human development index [7] and has some of the poorest health indicators in the world. Maternal mortality is about 835 per 100,000 live births [8], with a rate as high as 2525 per 100,000 live births in some regions [9]; and the under‐five child mortality rate is the sixth highest in the world [10]. Food insecurity is widespread which affects nearly half (47%) of the population [11]. The political situation has remained highly volatile in recent years, leading to chaos and paralysis of the health system throughout the country, including the capital, Bangui [12].

These health system challenges compound the capacity for CAR to ensure patients receive and adhere to ART. Sustained viral load suppression is needed to reduce HIV transmission and requires consistent adherence to ART in order to reduce AIDS‐related morbidity and mortality [13, 14, 15, 16, 17]. To date, retention to ARV treatment has not been well characterized in CAR. The objective of this study was to determine the rate of lost‐to‐follow‐up (LTFU) and to investigate factors associated with LTFU among people living with HIV (PLHIV) receiving ART in CAR.

## 2 METHODS

### 2.1 Study setting

The CAR has a population of about 6.1 million people. The estimated HIV prevalence in the CAR is 2.7% in 2022 (95% CI 2.2−3.3) [18] which represents approximately 88,000 PLHIV, of whom 55,345 (62.9%) have initiated ART [18]. The health sector in CAR has been organized into seven health regions, with most of the population living in health region 7 which includes the capital Bangui and surrounding districts, followed by the south‐western regions (health regions 1, 2 and 3).

**Figure 1:**
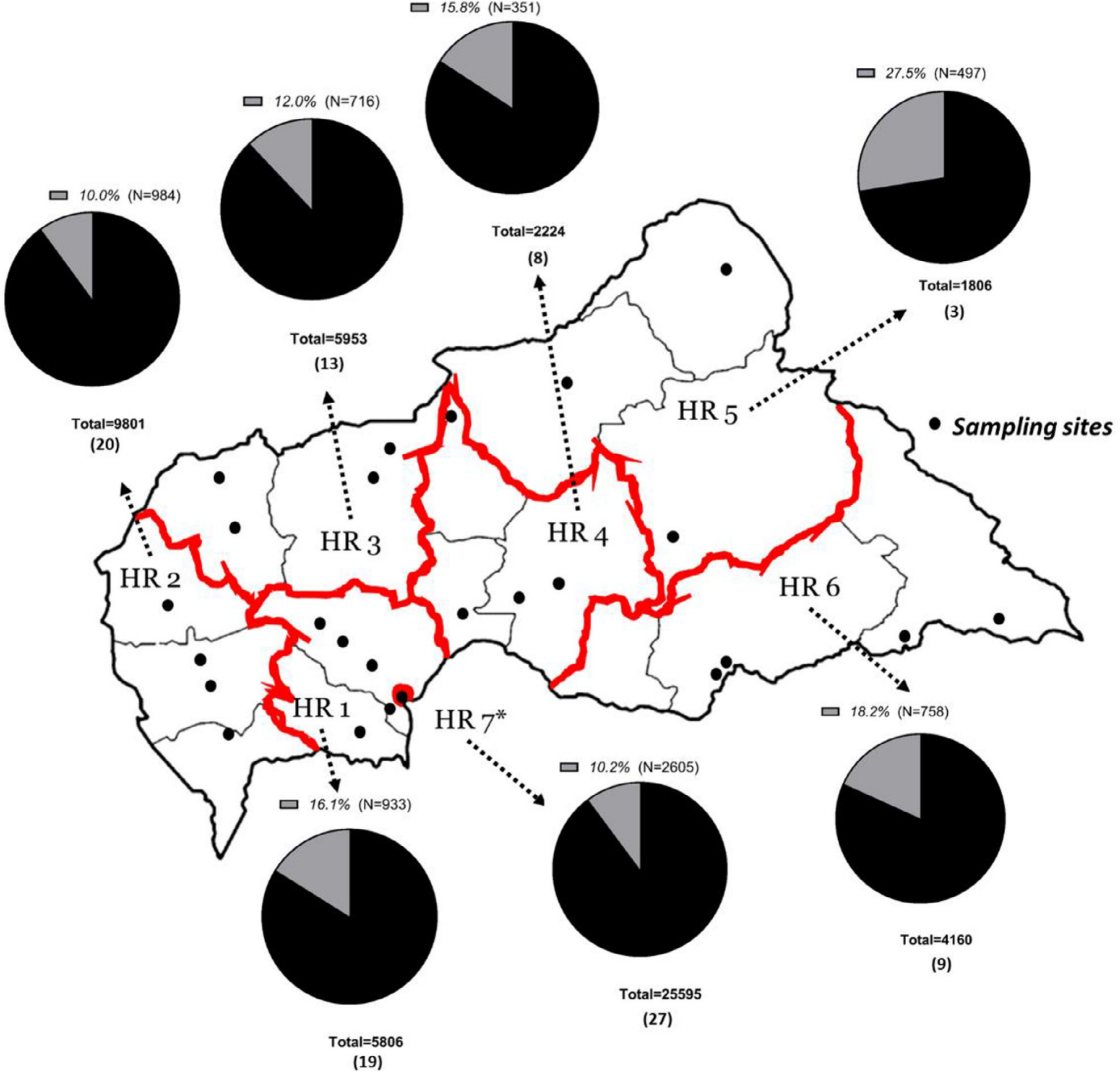
Health regions (here “HR”) of the CAR, number of PLHIV enrolled in care in 2021 and % included in the study sample.^1^ * Health region 7, centred around the capital city Bangui, includes 18 sampling sites. 1 In brackets, we indicate the number of ARV refill centres in each health region.

The distribution of the number of PLHIV receiving ART across 99 dispensing centres by health region are shown in Figure 1. Of note, health region 7 includes 27 ART refill centres which provides treatment for 46.2% of the national PLHIV cohort, whereas health region 5 covers only three centres and provides care for only 3.3% of PLHIV receiving ARV.

### 2.2 Study design and population

A retrospective cohort study was conducted using data from patients being managed at 42 representative ART dispensing sites in the seven health regions of CAR. Specifically, health centres that managed at least 200 patients were targeted for study inclusion. These ART dispensing sites represent about 80% of the total PLHIV cohort in CAR. However, centres with lower PLHIV cohorts were also included at convenience (i.e. if they were located along the routes to reach the primarily chosen centres). At last, 15% of eligible patients were randomly selected from patient records in selected centres. The study period spanned from 1 January 2019 to 31 December 2021. Patients were eligible for analysis if they initiated ART between January 2019 and September 2021 and were ≥13 years old, therefore, patients had at least 90 days of follow‐up to the end of the study period on 31 December 2021 (cohort close‐up date). Participants were censored at the first LTFU event. The test‐and‐treat strategy to HIV/AIDS was implemented in CAR in 2018, therefore, the date of first‐line ART initiation is the same as the date of HIV diagnosis and was considered the index date. An additional geospatial analysis was conducted with the cohort in the capital, Bangui, to test if PLHIV taking ART were attending the most accessible ARV refill centres.

### 2.3 Data collection

The following de‐identified data were extracted from the patients’ drug dispensing records for analysis: gender, age at ART initiation, body mass index (BMI), distance to healthcare facility, health region, WHO Clinical Stage (I−IV) at ART initiation, date of ART initiation and attendance dates for ART refills. As per standard of care, ART refills were scheduled monthly or multi‐month depending on the length of the ARV dispensed refill given to the patients when they attended clinic.

### 2.4 Outcome definitions

An LTFU event was defined as a failure of a patient to attend a scheduled ART refill appointment for at least 90 days (i.e. three consecutive monthly refill appointments missed) [19]. Retention to treatment was defined as not having had any LTFU event during the follow‐up period. Return to care (i.e. reengagement with ARV treatment) was defined as a return to an appointment following an LTFU event (i.e. defined as a gap between refill appointments of >90 days).

### 2.5 Statistical methods

Descriptive summary statistics (mean, median, standard deviation [SD], interquartile range for continuous variables; and number and proportion for categorical variables) was used to describe patient characteristics.

Chi‐squared test or Student's *t* test were used to compare categorical or continuous variables, respectively, or their non‐parametric alternatives when necessary (Fisher's exact test or Wilcoxon test) and sign rank test for paired data. Probability of retention in ARV care was estimated using Kaplan‐Meier (KM) survival methods. KM curves were plotted and cumulative probabilities at 6, 12 and 24 months following index date were estimated. Patients were censored if they were LTFU. Attrition from ARV care was based on a minimum follow‐up time of 6, 12 and 24 months. The proportion of attrition patients correspond to the number of patients not‐maintaining ARV treatment at the end of the studied time‐lapse. Smoothing using spline extrapolation with eight knots were estimated to identify trends and peaks of LTFU rates during the follow‐up period. Rates of LTFU per 1000 persons‐month and 95% confidence interval (CI) were estimated. To evaluate factors associated with LTFU, Cox regression models were constructed. Variables significant in univariate analysis were entered into a multivariate model; and hazard ratios (HR) and 95% CI were calculated. A *p*‐value ≤0.05 was considered statistically significant.

To map the residence of PLHIV in the capital Bangui, ARV refill centres and the Euclidean distance between them, we employed geographic information systems using ArcGIS 10.8.1 software.

### 2.6 Ethical issues

All data collected were de‐identified and anonymized by assigning a unique study identifier. Consent from patients was waived as data were anonymized before analysis. The study received ethical approval from the CAR Ministry of Health and Population's Comité National d’Éthique (reference number 074/MSP/DIRCAB/DEGELM/LMT).

## 3 RESULTS

### 3.1 General description

Table 1 describes the study sample overall and by health region. A total of 6844 ART patients met study inclusion criteria for analysis, of whom 41.3% (*N* = 2825) initiated ART care in 2019, 36.6% (*N* = 2507) in 2020 and 22.1% (*N* = 1512) in 2021 (up to September). Overall, the mean age at ART initiation was 35.3 years (SD 10.5), and two‐thirds were females (67.5%) which were younger than males (33.8 vs. 38.5 years; *p*<0.001). About 18% of patients were <25 years at ART initiation. About 28% of patients did not have WHO Stage data available. Among those with stage recorded, one‐quarter (27.3% = 1343/4920) had WHO Advanced Stage (III−IV). Most patients with BMI data available were underweight (i.e. suggestive of malnutrition) with 46.6% (= 1105/2372) having a BMI < 18.5 kg/m^2^. Most patients lived within <5 km of the health centre (42.6% = 2704/6328).

**Table 1:** Patient characteristics overall and by health region^a^

	Overall		Health region 1		Health region 2		Health region 3		Health region 4		Health region 5		Health region 6		Health region 7	
*N*	6844		933		984		716		351		497		758		2605	
**Sex**																
Females	4617	67.5%	656	70.3%	639	64.9%	473	66.1%	240	68.4%	329	66.2%	502	66.2%	1778	68.3%
Males	2222	32.5%	276	29.6%	344	35.0%	243	33.9%	111	31.6%	166	33.4%	256	33.8%	826	31.7%
Missing	5	0.1%	1	0.1%	1	0.1%	0	0.0%	0	0.0%	2	0.4%	0	0.0%	1	0.0%
**Mean age (standard deviation) (years)**	35.3	(10.5)	35.7	(10.4)	33.16	(9.6)	34.6	(9.9)	33.3	(10.4)	32.8	(10.9)	36.5	(11.2)	36.6	(10.4)
**Age category (years)—*n* (%)**																
13−25	1232	18.3%	151	17.0%	230	23.5%	126	18.0%	90	26.2%	144	29.1%	127	17.0%	364	14.1%
26−35	2472	36.7%	330	37.2%	389	39.7%	292	41.7%	124	36.2%	164	33.1%	256	34.3%	917	35.4%
36−45	1939	28.8%	258	29.1%	245	25.0%	188	26.9%	85	24.8%	128	25.9%	219	29.4%	816	31.5%
≥ 46	1097	16.3%	149	16.8%	116	11.8%	94	13.4%	44	12.8%	59	11.9%	144	19.3%	491	19.0%
Missing	104	1.5%	45	4.8%	4	0.4%	16	2.2%	8	2.3%	2	0.4%	12	1.6%	17	0.7%
**WHO Stage—*n* (%)**																
I−II	3577	72.7%	463	80.7%	550	74.7%	472	76.9%	116	74.4%	308	63.0%	466	86.3%	1202	66.4%
III−IV	1343	27.3%	111	19.3%	186	25.3%	142	23.1%	40	25.6%	181	37.0%	74	13.7%	609	33.6%
Missing	1924	28.1%	359	38.5%	248	25.2%	102	14.2%	195	55.6%	8	1.6%	218	28.8%	794	30.5%
**Median BMI (interquartile range)**	19 (17−21)		19 (17−21)		18.5 (17−21)		20 (18−22)		18 (17−20)		18 (6−20)		18 (17−20)		19 (17−22)	
**BMI (kg/m^2^)—category—*n* (%)**																
≥18.5	1267	53.4%	59	55.7%	297	50.0%	237	72.7%	35	42.7%	136	41.6%	105	43.9%	398	57.0%
<18.5	1105	46.6%	47	44.3%	297	50.0%	89	27.3%	47	57.3%	191	58.4%	134	56.1%	300	43.0%
Missing	4472	*65.3* *%*	827	*88.6%*	390	*39.6%*	390	*54.5%*	269	*76.6%*	170	*34.2%*	519	*68.5%*	1907	*73.2%*
**Distance for health centre (km). *n* (%)**																
<5	2704	42.6%	249	33.7%	518	53.6%	296	44.3%	145	70.1%	218	44.0%	288	39.3%	990	39.3%
5−15	2344	37.0%	175	23.7%	142	14.7%	145	21.7%	11	5.3%	137	27.7%	319	43.6%	1415	56.2%
>15	1280	20.4%	316	42.7%	306	31.7%	227	34.0%	51	24.6%	140	28.3%	125	17.1%	115	4.6%
Missing	516	7.5%	193	20.7%	18	1.8%	48	6.7%	144	41.0%	2	0.4%	26	3.4%	85	3.3%

Abbreviation: BMI, body mass index.

^a^ Percentages are calculated over the total number of participants with data available (not‐missing).

Generally, patient characteristics were broadly similar across health regions by age and sex. In comparison to other health regions, health region 6 had the lowest proportion of patients with WHO Advanced Stage III and IV (13.7%) and health region 7 had the highest (33.6%). Health region 5 had the highest proportion of patients who were underweight with a BMI <18.5 kg/m^2^ (58.4%), whereas health region 3 had the lowest (27.3%). Participants with missing were similar in terms of age, sex distribution and year of initiation to the rest of the sample (*p*>0.05).

### 3.2 Retention outcomes, LTFU rates incidence and associated risk factors

**Figure 2:**
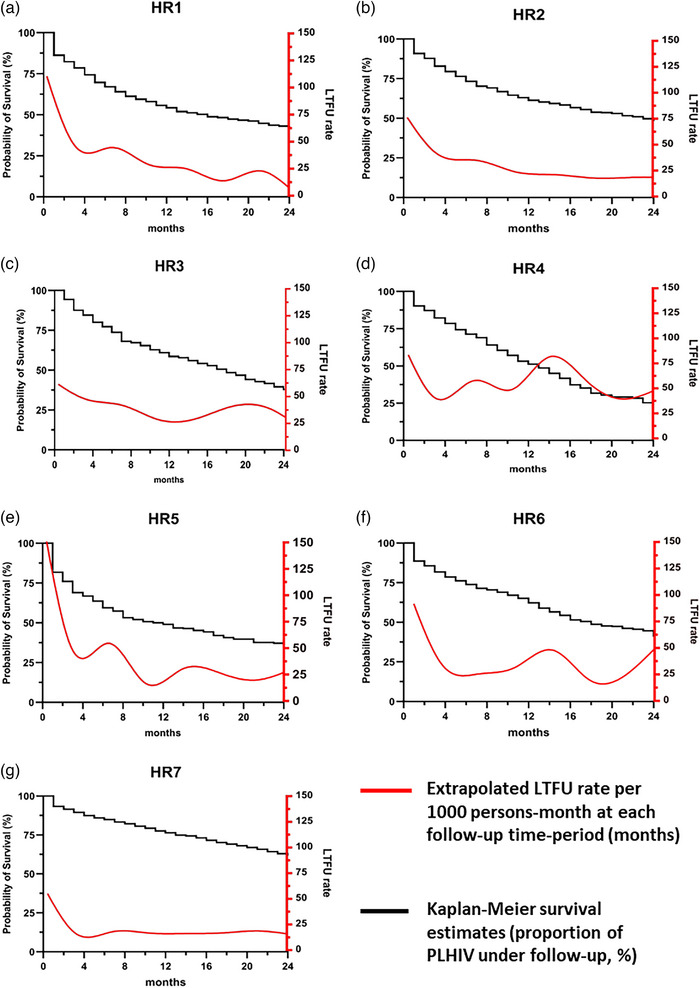
(a−g) Kaplan‐Meier estimates (black lines) of the cumulative probability of patient retention in antiretroviral treatment (ART) by health region (here, “HR”) and smoothed rate estimates of lost to follow‐up stratified by health region (red lines). Rates are expressed in 1000 persons‐month.

In total, we observed 7298 person‐years of follow‐up and 2874 LTFU events. Table 2 summarizes the rates of LTFU, return to care and retention in ART care and attrition. Overall, 42% of patients were LTFU during the study period and this ranged from 31.4% in health region 7 to 58.1% in health region 4. However, 23.2% returned to care after LTFU and this ranged from 12.3% in health region 3 to 28.1% in health region 7. Median time to return to care after LTFU was 3.5 months overall. Retention in care was estimated at 75.7% (CI 74.7−76.7), 64.2% (CI 63.0−65.5) and 48.3% (CI 46.8−49.8) at 6, 12 and 24 months. Retention was lowest in health region 4 at 24 months (24.5%; CI 18.6−30.7) and highest in health region 7 (61.6%; CI 59.0−63.4). Attrition estimations was of 25.2%, 35.8% and 45.8% for patients with at least 6, 12 and 24 months of follow‐up time, respectively. Similar trends were observed across all regions. Figures 2a−g show the KM projections of patient retention and smoothed rate estimates of LTFU by health region.

**Table 2:** Lost to follow‐up, return to care, retention in care estimation and ART attrition estimations

	Overall		Health region 1		Health region 2		Health region 3		Health region 4		Health region 5		Health region 6		Health region 7	
*N*	6844		933		984		716		351		497		758		2605	
**LTFU (%)**	2874	42.0%	440	47.2%	427	43.4%	366	51.1%	204	58.1%	264	53.1%	355	46.8%	818	31.4%
**Return to care after LTFU**	666	23.2%	94	21.4%	88	20.6%	45	12.3%	52	25.5%	60	22.7%	97	27.3%	230	28.1%
**Median time (months) to return to care after LTFU (interquartile)**	3.5	(1.5−6.5)	2.5	(3.5−5.5)	4.5	(2.5−6.5)	2.5	(1.5−3.5)	4.5	(2.5−7.5)	3.5	(1.5−10.5)	3.5	(1.5−5.5)	3.5	(1.5−7.5)
**Retention in ARV care (%) (Kaplan‐Meier estimation)** ^a^																
Retention (%) 6 months	4764	75.7%	562	66.7%	664	73.0%	508	73.6%	224	71.0%	261	58.8%	507	73.6%	2038	84.8%
Retention (%) 12 months	3186	64.2%	307	53.6%	446	60.8%	317	58.2%	133	50.6%	140	48.2%	314	61.7%	1529	76.1%
Retention (%) 24 months	1228	48.3%	126	41.6%	187	48.0%	122	37.3%	29	24.5%	66	34.7%	124	40.6%	574	61.6%
**Attrition (%) from ARV care based on minimum follow‐up time** ^b^																
Attrition (%) with 6 months follow‐up (*N* = 5371)	4017	25.2%	446	38.9%	580	27.0%	422	21.6%	181	33.2%	194	45%	388	29.6%	1806	15.5%
Attrition (%) with 12 months follow‐up (*N* = 4706)	3022	35.8%	309	50.7%	427	37.2%	302	38.5%	125	46.6%	131	57%	293	39.8%	1435	23.8%
Attrition (%) with 24 months follow‐up (*N* = 2990)	1241	45.8%	132	58.1%	182	45.8%	111	53.7%	42	63.2%	70	55%	121	52.5%	583	33.2%

Abbreviation: LTFU, lost to follow‐up.

^a^ Numbers shown exclude patients censored at 6, 12 or 24 months as appropriate.

^b^Attrition estimates include patients with a minimum follow‐up reporting period of 6, 12 or 24 months for each respective time‐lapse. Therefore, the proportion (%) of patients shown correspond to the number of patients not maintaining ARV treatment at the end of the reporting period over those under ARV at 6, 12 or 24 months before the cut‐off.

Table 3 describes the rates of LTFU and crude and adjusted hazard ratio estimates of factors associated with LTFU. The rate of LTFU was estimated at 32.8/1000 persons‐month (CI 31.6−34.0). The highest rate of LTFU was in health region 5 (56.6 per 1000 person‐months; CI 50.2−63.8) and 6 (56.4 1000 person‐months; CI 49.2−64.7) and was the lowest in health region 7 (21 per 1000 person‐months; CI 19.6−22.5). In univariate analysis, being male, younger age (age ≤35 years), having an underweight BMI <18.5 kg/m^2^, living >15 km away from the healthcare facility and attending a healthcare facility other than health region 7 were associated with higher rate of LTFU.

**Table 3:** Rate of LTFU and factors associated with LTFU by patient characteristics

		No. of person‐years follow‐up	No. of LTFU events	Rate per 1000 person‐months		Hazard ratio (HR)			Adjusted Hazard ratio (aHR)^a^		
	*N*			Rate	95% CI	HR	95% CI	*p*‐value	aHR	95% CI	*p*‐value
**Sex**											
Females	4617	4995	1901	31.7	30.3−33.2	1 (ref)			1 (ref)		
Males	2222	2300	969	35.1	33.0−37.4	1.1	1.0−1.2	0.02	1.33	1.2‐1.5	<0.001
**Age category (years)—*n* (%)**											
13−25	1232	1202	563	39	35.9−42.4	1.31	1.2−1.5	<0.001	1.46	1.1‐1.9	0.002
26−35	2472	2650	1077	33.9	31.9−36.0	1.16	1.0−1.3	0.009	1.25	1.0‐1.6	0.04
36−45	1939	2119	743	29.2	27.2−31.4	1.01	0.9−1.1	0.9	1.1	0.9‐1.4	0.4
≥ 46	1097	1238	429	28.9	26.3−31.8	1 (ref)			1 (ref)		
**WHO Stage—*n* (%)**											
I−II	3577	3820	1510	32.9	31.3−34.6	1 (ref)	0.9−1.2	0.2	1 (ref)		
III−IV	1343	1363	574	35.1	32.3−38.1	1.06			0.96	0.8−1.1	0.6
**BMI (kg/m^2^)—category—*n* (%)**											
≥18.5	1267	1410	482	28.5	26.1−31.1	1 (ref)			1 (ref)		
<18.5	1105	1041	468	37.5	34.2−41.0	1.25	1.1−1.4	0.001	1.13	1.0−1.3	0.07
**Distance to healthcare facility (km). *n* (%)**											
< 5	2704	3031	1103	30.3	28.6−32.2	1 (ref)			1 (ref)		
5−15	2344	2581	926	29.9	28.0−31.9	0.98	0.9−1.1	0.6	1.15	1.0−1.4	0.09
> 15	1280	1234	584	39.4	36.4−42.8	1.25	1.1−1.4	<0.001	1.05	0.9−1.3	0.6
**Health region (HR)**											
HR 1	933	824	440	44.5	40.5−48.9	1.96	1.7−2.2	<0.001	2.23	1.6−3.2	<0.001
HR 2	984	1040	427	34.2	31.1−37.6	1.51	1.4−1.7	<0.001	1.73	1.4−2.2	<0.001
HR 3	716	759	366	40.2	36.3−44.5	1.83	1.6−2.1	<0.001	1.56	1.2−2.0	0.001
HR 4	351	301	204	56.4	49.2−64.7	2.37	2.0−2.8	<0.001	3.31	2.0−5.4	<0.001
HR 5	497	389	264	56.6	50.2−63.8	2.41	2.1−2.8	<0.001	2.91	2.3−3.6	<0.001
HR 6	758	743	955	39.8	35.9−44.2	1.8	1.6−2.0	<0.001	2.52	2.0−3.2	<0.001
HR 7	2605	3242	818	21	19.6−22.5	1 (ref)			1 (ref)		

Abbreviation: BMI, body mass index.

^a^ Adjusted hazard ratio model was adjusted for all factors (sex, age, WHO Stage, BMI, distance to healthcare facility and health region).

Risk factors associated with an LTFU event after adjusting for these factors were being male (adjusted hazard ratio [aHR], 1.33; 95% CI 1.1−1.5), being < 25 years old (aHR 1.46; CI 1.1−1.9) and between 26−35 (aHR 1.25; CI 1.0−1.6) compared to patients older than 45 years, living outside health region 7 (pooled aHR 1.83; CI 1.6−2.3) and fulfilling criteria for underweight (aHR 1.13; CI 1.0−1.3). Living further than 15 km from the refilling centre (HR 1.25; CI 1.1−1.4) at ART initiation was not associated with increased LTFU risk after adjustment.

### 3.3 Geographical analysis of health‐seeking behaviour in the capital Bangui

**Figure 3:**
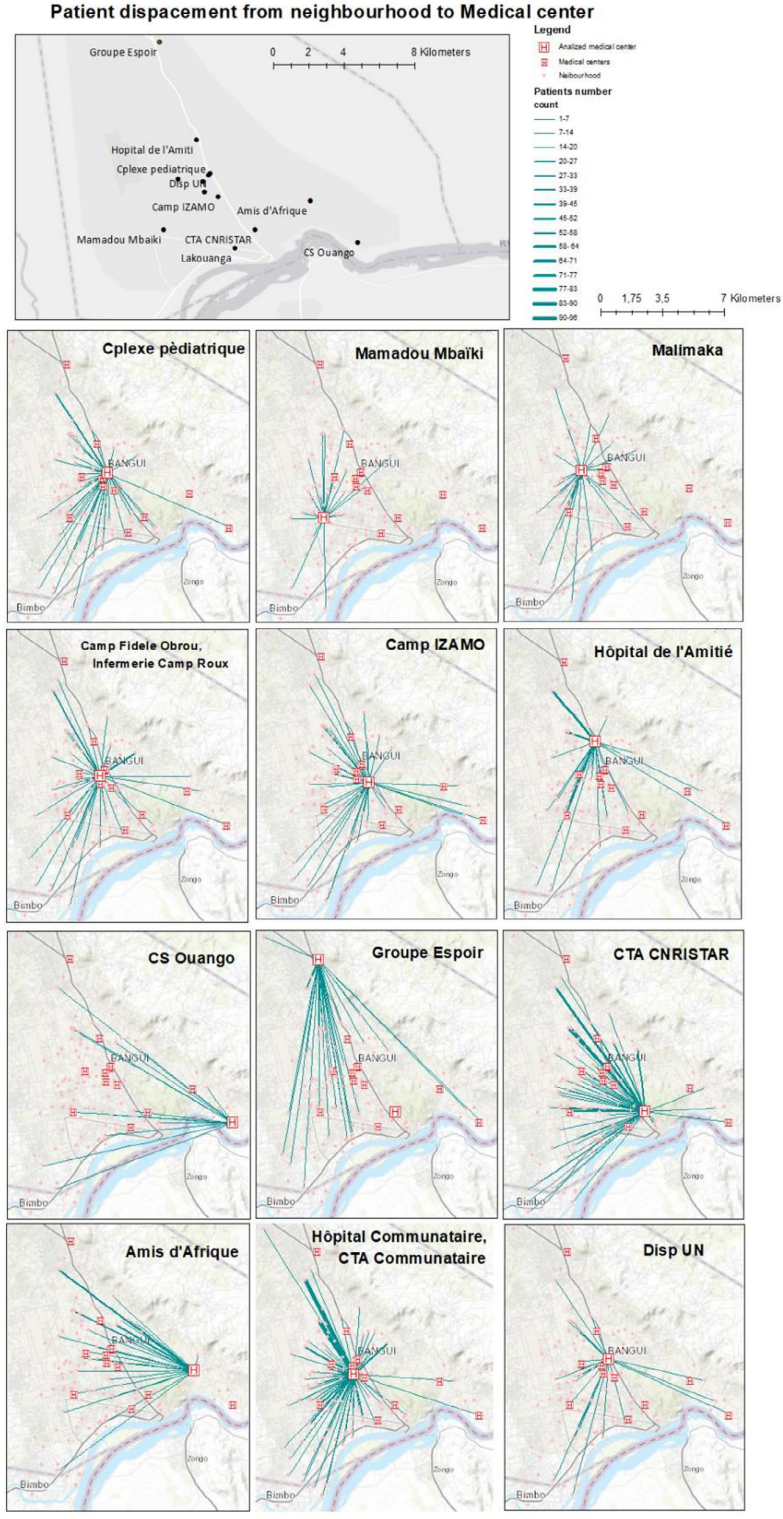
Geospatial localization of persons living with HIV (PLVIH) living in Bangui under antiretroviral treatment (ART) respective to the refill centres; drafted lines link patients or cluster of patients with the respective refill centre.^1^ 1. Each start line indicates the centroid of the neighbourhood where patients are living. Lines represent the Euclidean distance between the ARV refill centre where these patients are followed up and the neighbourhood of residence. Lines width correlates by the number of patients included in each node (see the legend).

Figure 3 shows the geographical analysis of health‐seeking behaviour in the capital (Bangui). The analysis was able to determine the neighbourhood of residence of 72.8% (= 1897/2605) of participants living in the health region 7. According to the Euclidean estimation between neighbourhood of residence and ARV dispensing centres, the median distance to the ARV refill centre was 3.6 km (IQR 2.2−5.5), whereas the median distance to the closest centre was of 1.6 km (IQR: 0.8−1.8; *p*<0.001 for paired data). Only 214 (11.3%) of georeferenced patients attended the closer ARV refill centre. The geospatial localization of participants respective to the refill centres shows a clear exocentric pattern (Figure 3).

## 4 DISCUSSION

We observed a suboptimal retention in treatment rate of PLHIV in the CAR with rate which was lower than that found for other cohorts in sub‐Saharan Africa (64.2% vs. 76.8% retention at 12 months, respectively) [20]. However, a sizable proportion of patients who were LTFU returned to care (23.2%). Individual‐level risk factors associated with LTFU were similar to those observed in other sub‐Saharan cohorts (young, being male, living in non‐urban areas, undernutrition) [21, 22, 23, 24, 25, 26, 27, 28, 29, 30]. The reasons behind these risk factors have been previously discussed and are consistent with other studies conducted in sub‐Saharan Africa. For instance, advanced HIV disease at diagnosis has been associated with poor retention in care, and a low BMI score has been considered a surrogate for advanced disease in the absence of CD4 determination [31]. However, we hypothesize that in the CAR this could be due to a reverse causality effect. People with food insecurity, and therefore, at risk of malnutrition, prioritize the use of scarce resources to seek food or to work on farms rather than to attend ARV refill centres [32, 33]. This might explain the higher rate of LTFU among those who have a lower BMI in our study. Furthermore, the CAR has one of the region's poorest road networks, making moving around the country extremely difficult (and this situation is worsened by the insecurity conditions), and the costs associated with transportation among the poorest populations may be overwhelming [33]. Another causal pathway between undernutrition and non‐adherence (and retention) is the fact that pill‐taking can be difficult on an empty stomach (e.g. due to nausea). According to care providers, this is a common complaint among PLHIV in the CAR and has been observed elsewhere [34]. Considering that food insecurity is widespread in the CAR [11], this condition may well be an important driver of LTFU.

A second particular driver of LTFU may be the high degree of stigma experienced by PLHIV in CAR [35], a well‐documented impediment to retention in care [3, 36]. In the local lingua franca (Sango), there is a specific term commonly used to indicate a PLHIV (“*kanga na pelle”)*, which means “man walking to death” [37]. This expression denotes the fact that such persons are systematically shunned and denied basic rights in CAR. Patients might abandon treatment due to fear of being stigmatized or they will seek care further away from the closest healthcare facility to their home, as suggested by the geospatial analysis of patients in Bangui (Figure 3). These observations are consistent with other studies that show PLHIV tend to travel farther to the health centres to maintain confidentiality [38, 39]. It should be noted that PLHIV have the right to freely choose their care facility in CAR. Otherwise, we would expect that patients living further than 15 km are less compliant than those living closer to the ARV refill centres. One of the explanations of this lack of association in the adjusted analysis is that patients tend to avoid the closer refill centres due to stigma. Or alternatively, those living far away from the refill centres did not have even the possibility to access to HIV screening and care. In other words, those accessing ART who live more than 15 km were only particular individuals with higher motivation or with more resources to reach the refill centres or are seeking for confidentiality. This health‐seeking behaviour pattern may hamper the expected benefits of any decentralized strategy [40].

Lack of access and mobility by any reason is a prominent risk factor for treatment interruption throughout sub‐Saharan Africa [41]. This might explain the high instability of the cohorts from regions 4 and 5 which are the more conflict‐prone in CAR [12], as suggested by the irregular shape of smoothed rate estimates of LTFU compared to other regions (Figures 2a−g). It is of interest to note that in health region 4 most PLHIV who are receiving ART (up to 70%) live within 5 km of their refill centres (Table 1), in stark contrast with other regions. Given that this area is the most unstable region in CAR, this suggests that only people living near their refill centre feel safe enough to access ARV treatment regularly. Of note, LTFU associated with voluntary or forced mobility might be an indicator of further transmission of HIV [42, 43, 44, 45]. As of April 2023, the internally displaced population in CAR due to violence is calculated to be around 489,000, while some 742,000 citizens are refugees in neighbouring countries [12]. Another prevalent factor in CAR is the widespread presence of informal mining sites which attract mainly young and mobile populations [46] and might be an important driver of LTFU there and are in turn hotspots of HIV transmission [47, 48, 49, 50]. Given these numbers, the interplay between mobility, underlying factors, HIV risk acquisition and LTFU deserves further consideration in CAR. The key role played by population movements in the epidemiology of HIV has been examined in detail elsewhere for Namibia [51] and Botswana [52, 53].

There is no question that mitigating some contextual factors such as achieving a degree of politico‐social stability would positively impact on HIV prevention and control. Unfortunately, this is not likely to happen in the CAR in the short‐term. In the meantime, certain actions could be implemented. Provision of ART to displaced people in humanitarian crisis has been demonstrated to be feasible [54, 55]. The non‐governmental organization Doctors Without Borders (MSF) has tested several novel strategies for the administration of ARVs in conflict situations with a certain degree of success in terms of viral load suppression [56, 57]. The integration of food distribution programmes with ARV provision makes sense given the high prevalence of food insecurity [11] and its links with LTFU [58]. This supplementation programme could increase the success of therapy overall and decrease AIDS‐related and non‐AIDS‐related mortality among PLHIV [59]. Finally, long‐lasting injectable ARVs have demonstrated their effectiveness in recent clinical trials in sub‐Saharan Africa [60] and may play an important role in the future [61], but the feasibility of implementation in contexts like the CAR still needs to be evaluated.

Various limitations need to be considered. Firstly, retention to care (picking up the ARV pills) might not fully correlate with adherence to ARV (effective regular intake of ARV pills). A significant number of PLHIV might be in retention to care but are not adherent to ART, as reported by care providers. Secondly, there is no electronic health record system which would allow cross‐checks of patients attending health centres. In addition, there is no reliable vital data registry system in the country. Therefore, the proportion of patients with LTFU who had in fact died, or had self‐transferred to another clinic or whose transfer was not registered could not be determined in this study and might result in a larger number of LTFU. Furthermore, LTFU incidence in a given ARV refill centre may be driven by clients seeking confidentiality (as suggested by the geospatial analysis), or other factors that have not been evaluated, like the quality of the service provided.

Causes underlying death or transfers may be similar to those underlying LTFU (fear of stigma and any mobility cause by itself). In a pooled analysis from cohorts in sub‐Saharan Africa, Zürcher et al. found that 34.2% of LTFU were due to unreported deaths and 23.9% undocumented transfers [62]. Similarly, Wilkinson et al. found that 18.6% of undocumented transfers and 38.8% of deaths in those LTFU [63]; and Chammartin et al. reported 14.8% of unnoticed transfers and 21.8% of deaths among those who could be traced [64]. However, these analyses did not consider the time effect, which may increase the rate of mortality among those LTFU over time since they abandoned ARV treatment and died of an AIDS‐related cause. Therefore, mortality could be considered as the fatal outcome of LTFU rather than the cause of LTFU in most of these cases. Overall, these results point to an over‐estimation of the number of PLHIV on ART as well as underestimates of retention in care rates in our study.

The study had some bias in the sampling frame process. Centres with smaller cohorts were not included, especially those residing in the rural countryside. These centres tend to have higher rates of LTFU [28]. Finally, a handful of patients under 6 multi‐month dispensing regimen could not have been monitored until the end of the follow‐up period. Not significant differences with the current results were found when considering at last 6 months of follow‐up (cohort censorship on 30th June 2021, data not shown).

## 5 CONCLUSIONS

This study underlines the difficulties involved in ARV in complex settings and the interplay between poor patient retention, mobility and other contextual factors like social unrest, stigma and food insecurity, but also the need for tailored programming and interventions to mitigate these factors and ensure the provision of ARV to PLHIV in such difficult contexts. Previous studies have demonstrated the potential of various strategies to address the kinds of problems particular to conflict‐fraught areas like the CAR.

## COMPETING INTERESTS

The authors declare no competing interests.

## AUTHORS’ CONTRIBUTIONS

GT, JI, CY, XV: Study design, data collection, analysis, article writing and revision; CN, PPL, LG, AH, SR: Administrative support, logistics, funding, supervision, article revision; SR, RM, MCB, LBM, PRM, KR, AS, FR: Data collection, supervision, article revision; LM‐P, EG, CM, SO: Data provision, article revision; MAN‐A: Data analysis, article revision; SH: Supervision, funding, article revision. All authors revised and approved the final version of the manuscript to the JIAS.

## FUNDING

The study was supported by Global Fund grant recipient CAF‐C‐CRF.

## Data Availability

Data could be available upon request to the Ministry of Health and Population from the Central African Republic after ethical clearance.
